# ukbpheno v1.0: An R package for phenotyping health-related outcomes in the UK Biobank

**DOI:** 10.1016/j.xpro.2022.101471

**Published:** 2022-06-17

**Authors:** Ming Wai Yeung, Pim van der Harst, Niek Verweij

**Affiliations:** 1 University of Groningen, University Medical Center Groningen, Department of Cardiology, 9700 RB Groningen, the Netherlands; 2 Department of Cardiology, Division of Heart & Lungs, University Medical Center Utrecht, University of Utrecht, Utrecht, the Netherlands

**Keywords:** Bioinformatics, Health Sciences, Systems biology

## Abstract

The complexity and volume of data associated with population-based cohorts means that generating health-related outcomes can be challenging. Using one such cohort, the UK Biobank—a major open access resource—we present a protocol to efficiently integrate the main dataset and record-level data files, to harmonize and process the data using an R package named “ukbpheno”. We describe how to use the package to generate binary phenotypes in a standardized and machine-actionable manner.

For complete details on the use and execution of this protocol, please refer to Yeung et al. (2022).

## Highlights

• A protocol to efficiently process UK Biobank phenotypic data
• Reproducible and semi-automatic generation of binary health-related phenotypes
• Utilities with visualization to aid data exploration and phenotype definition
• Example analyses common among genetic epidemiological studies

## Abstract (editor highlights)

Publisher’s note: Undertaking any experimental protocol requires adherence to local institutional guidelines for laboratory safety and ethics.

## Graphical abstract

**Figure:**
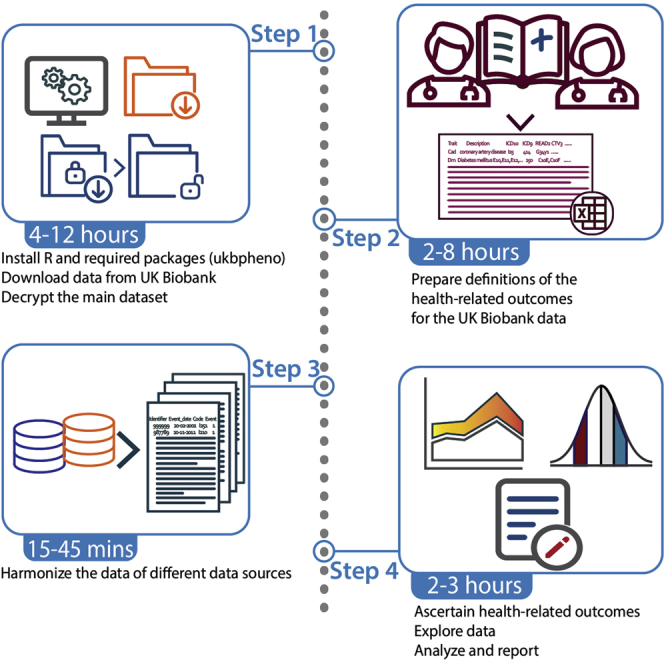

## Before you begin

The UK Biobank is a large-scale population cohort with in-depth collection of phenotypic and genetic data (Sudlow et al., 2015; Bycroft et al., 2018). While it is undoubtedly an invaluable resource for biomedical research, the magnitude of data volume, complexity of information collected as well as the longitudinal nature of data may pose a challenge for researchers to phenotype health related outcomes. The evidence supporting the diagnosis of a certain health outcome may be reported by the participants (during their visits to the assessment centers or online follow-ups) or via linkage to national registries, primary care and secondary care data. The self-report data at the assessment center is further divided into two categories, namely those reported during a nurse interview (Field 20001, 20002, 20003, 20004) and touchscreen (multiple fields) of which customized data-coding systems apply. Reliabilities and coverage of the source in terms of time period as well as proportion of cohort are also likely important factors to consider as well (Woodfield et al., 2015; Eastwood et al., 2016; UK Biobank, 2019). The extent of capture by a data source varies between health outcomes such as in the case of diabetes as reported previously by Eastwood and colleagues (Eastwood et al., 2016). An interactive phenotyping process, namely to be able to navigate and explore the data from various sources, is therefore desirable.

**Figure 1:**
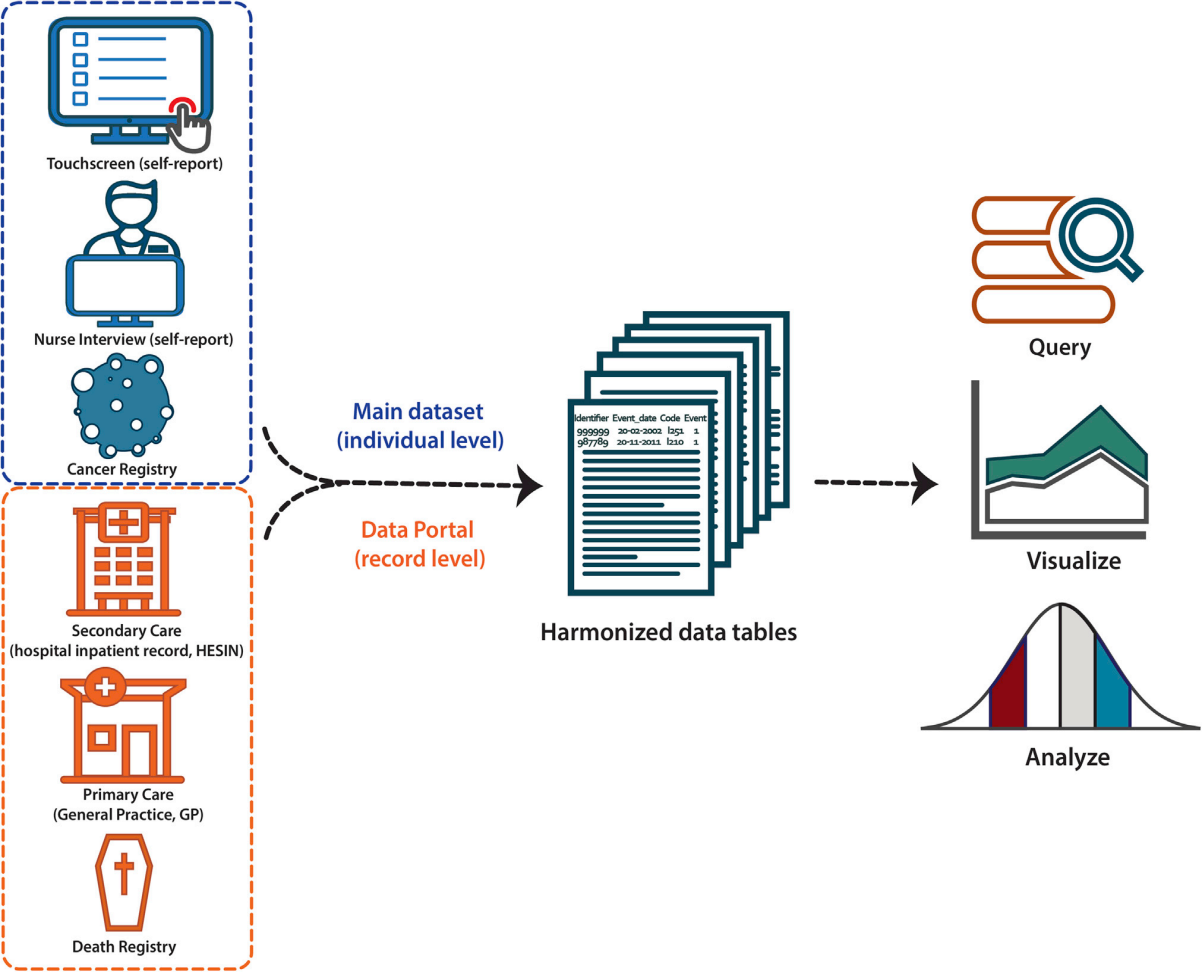
Capture of health-related outcome from multiple data sources in UK Biobank using ukbpheno

In line with the FAIR principle (Wilkinson et al., 2016), it is advantageous to transform the data of different sources into a consistent structure with machine readable data / metadata which improves the operability and reproducibility of the phenotyping. Automation of the phenotyping process also helps in reducing human errors. Therefore, we developed an open-access R package (ukbpheno, available at https://github.com/niekverw/ukbpheno). The package contains functionalities to harmonize data of various sources in a consistent manner accompanied by structured metadata. To allow interactive exploration, the package contains multiple functionalities to visualize the data and it can be run on typical workstations (high-performance computing cluster is not required). Figure 1 illustrates the main concept of data integration and transformation using the ukbpheno package.

**Figure 2:**
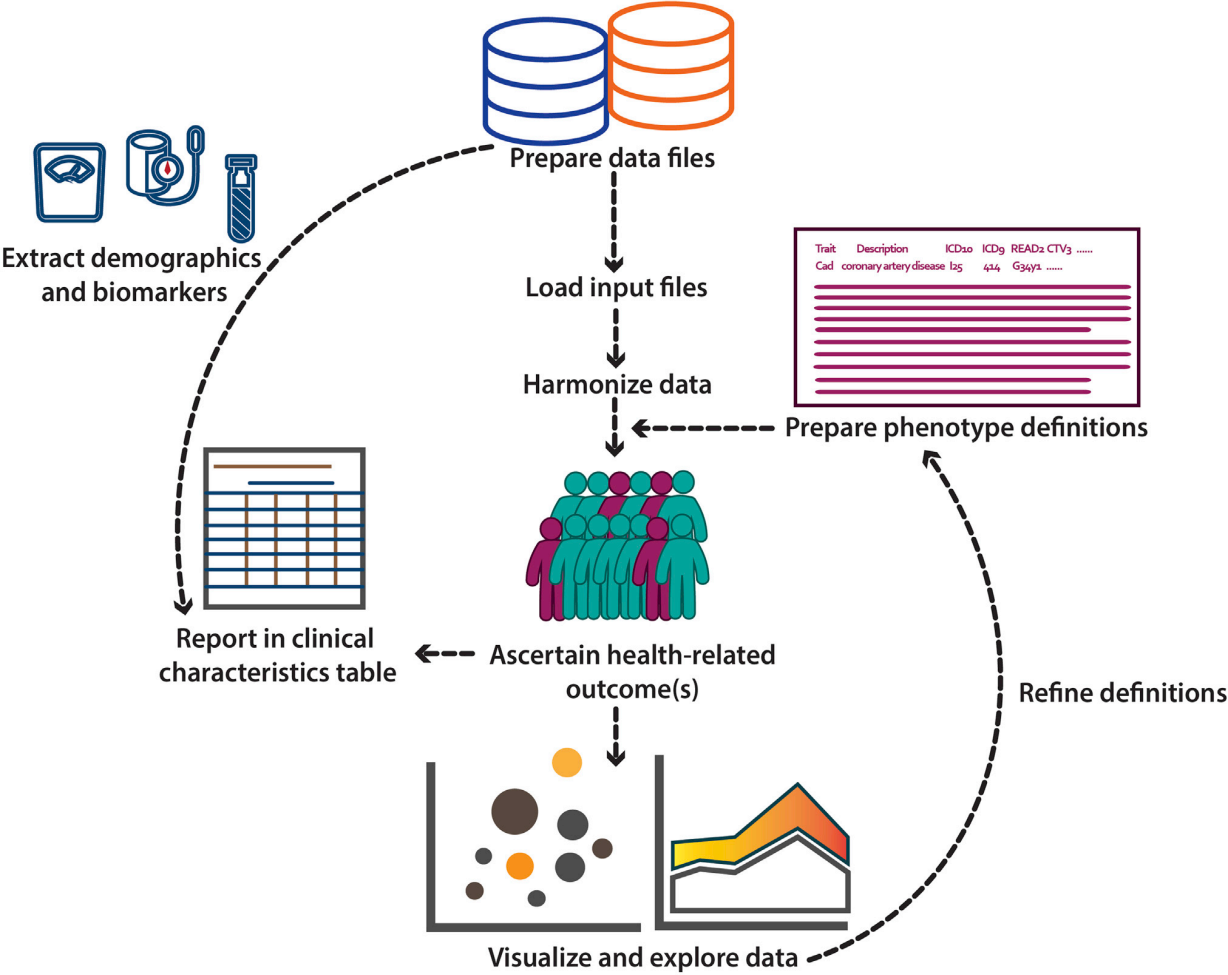
Overview of the workflow

In this protocol, we show how to download and process the data from the UK Biobank as well as to generate health related phenotypes with the use of ukbpheno. More specifically, we utilize the ukbpheno package to first harmonize various data into single episode format and then generate the phenotypes (Figure 2). We then demonstrate the use of package for in-depth exploration of the harmonized data with example visualizations and analyses. As an example, we demonstrate the ascertainment of type 2 diabetes, considering the rationales put forward by Eastwood and colleagues (outlined in S1 Appendix) (Eastwood et al., 2016). We use data from self-report sources (both touchscreen and nurse interview), secondary care data and primary care data to identify people with type 2 diabetes while ruling out other types of diabetes (gestational diabetes and type 1 diabetes) as well as use of metformin due to other conditions. In the second part we show how to automate the phenotyping process, namely to generate multiple cardiometabolic traits used in our previous studies (van der Harst and Verweij, 2018; Verweij et al., 2020; Benjamins et al., 2022; Yeung et al., 2022). The diagnosis codes used for these traits are shown in Table 1. With these phenotypes, we perform some example analyses including the generation of a clinical characteristic table and a survival analysis.

**Table 1:** Definitions of the cardiometabolic traits to be generated

Variable	ICD-9	ICD-10	OPCS-4	Self-reported fields	READ2	CTV3
Atrial fibrillation or flutter	4273	I48	K621, K622, K623	20002(1471, 1483)	G5730, G573., G5731, G573z, G5733, G5732, 793M0, 793M1, 793M3, 79345, 79348	XaEga, G573., G5730, G5731, G573z, X202R, X202S, Xa2E8, XaMmd, XaMmc, XaMrB, XaLgF, XaMrA
Coronary artery disease	414, 410, 412	I24, I25, Z955, I21, I22, I23, I252, Z951, Z955	K40, K41, K42, K43, K44, K45, K46, K49, K50, K75	20002(1075), 20004(1070, 1095, 1523),6150(1)	G34y1, G34.., G3..., ZV45L, G34z0, ZV458, 793G., 79280, 79281, 79282, 7928y, 7928z, 79292, 7929y, 7929z, 792.., 7A547, 793Gy, 793Gz, 79283	G34y1, XE0WG, XE2uV, XaC1g, XaG1Q, XaQiY, ZV458, G34.., X200b, Xa1dP, XaLgU, 79280, 79281, 79282, 7928y, 7928z, 79292, 7929y, 7929z, X00tT, X013N, XE0Em, XaLgZ, XaLga, XaMKE
Hypertrophic cardiomyopathy	4251	I421, I422		20002(1588)	G551.	G551., X201Y
Heart failure	428	I50, I110, I130, I132, Z941, T862	K02	20002(1076), 20004(1098)	14S3., G2101, G2111, G21z1, G232., G234., G58.., G5800, G5802, G5803, G5810, G582., SP084, SP111, G581., 1O1.., G583., ZV421	14S3., G2101, G2111, G21z1, G232., G234., G58.., G5800, G5802, G5803, G5810, G582., SP084, X202k, X202v, X202w, XE2QG, XaIpn, XaWyi, ZV421, X00y3
Type 2 diabetes^a^	25000,25002,25010,25012,25020,25022,25030,25032,25040,25042,25050,25052,25060,25062,25070,25072,25080,25082,25090,25092	E11		20002(1223)	C1001, C10F9, C10F., C1093, C1094, C1095, C1097, C10y1, C10z1, C10FJ, C109J, C1099, C10FD, C109D, C10FR, C10FK	C1001, C1011, C1031, C1071, C1021, C1072, C1093, C1094, C1095, C1097, C10y1, X40J5, X40J6, XaELQ, XaFWI, XaIrf, XaKyX, X40J5
Hypertension	402, 403, 404, 405, 401	I11, I12, I13, I15, O10, I10		20002(1065, 1072),6150(4),6177(2),6153(2),20003	G21.., G220., G221., G23.., G24.., G240., G240z, G241., G241z, G24z., L12.., G22.., G22z., G2z.., G2y.., G21z0, G20.., G20z., x01QX	G21.., G220., G221., G23.., G24.., G240., G240z, G241., G241z, G24z., L12.., XE0Uf, XE0Ug, G2z.., G2y.., Xa0lt, Xa3fQ, Xa0kX, XE0Uc, x01QX
Hyperlipidemia	272	E78		20002(1473),20003	C32.., Cyu8D, Cyu8E	X40Uu, XE11R, C32.., C32z., X40Wx

Variable definitions constructed using ICD-9, ICD-10, OPCS-4, READ2 and CTV3 codes as well as self-report data fields with disease- or procedure-specific codes between brackets are shown.

Abbreviations: CTV3, Clinical Terms Version 3; ICD, International Classification of Diseases; OPCS, Office of Population, Censuses and Surveys: Classification of interventions and Procedure.

a Cases with type 1 diabetes specific codes are excluded and controls with any diabetes related codes are excluded.

### Download input files from UK Biobank

**Timing: 4–12 h**

Health outcome information is collected in numerous ways. Individual-level data are stored in the main dataset which includes self-report outcomes during the nurse interview, data from cancer registry and death registry. Clinical variables and biomarkers which may be useful for defining a health outcome such as glycated hemoglobin are also stored in the main dataset. Record-level data from hospital inpatient data, primary care data, death registry as well as COVID-19 test results are available through the Data Portal. It is important to note the data from the Data Portal is updated more frequently than the main dataset. As a result, the data in the main dataset and the data from the Data Portal may not be updated at identical dates. Users should be aware of the source of the data and consult the Showcase for the corresponding censoring dates.

***Note:*** The UK Biobank data are an open access resource to any bona-fide researchers. The data analyzed in this protocol were obtained through application number 74395. Please refer to the following link to request access to UK Biobank data: https://www.ukbiobank.ac.uk/enable-your-research/apply-for-access.

1. Download and decrypt the main dataset.
  a. Follow the steps in section 2 of the data access guide (https://biobank.ctsu.ox.ac.uk/∼bbdatan/Accessing_UKB_data_v2.3.pdf) to obtain and decrypt the main dataset from the UK Biobank Data Showcase (Showcase) (https://biobank.ctsu.ox.ac.uk/crystal/download.cgi). Files needed in this step include:
    i. An MD5 Checksum to verify download (sent to principal investigator and authorized delegates).
    ii. A key file for decryption (sent to principal investigator and authorized delegates).
    iii. Utilities software “ukbmd5” and “ukbunpack” available on Showcase.
  b. Generate a metadata file (.html) of the decrypted main dataset (.enc_ukb) using utility “ukbconv” (available on Showcase).
    ukbconv ukbxxxxx.enc_ukb doc
  c. Generate a tab-separated file (.tab) of the decrypted main dataset (.enc_ukb) using utility “ukbconv”
    ukbconv ukbxxxxx.enc_ukb r
    **CRITICAL:** Do not run the accompanied ukbxxxxx.R script generated at this step. Only the tab separated file (.tab) is required.

2. Obtain the record-level data from Data Portal on Showcase (https://biobank.ndph.ox.ac.uk/showcase/).
  a. Click the “Login” on the top of the webpage which redirects users to the Access Management System.
  b. Once logged in, select “Projects” on the left panel of the Access Management System and view the relevant project (“View/Update”).
  c. Inside the project, select the “Data” tab to connect the Showcase in “logged-in” mode.
    i. Once in the Showcase, move to “Downloads” tab on the top of the webpage.
    ii. In the “Downloads” page, researchers with access to record-level data will see a tab “Data Portal” next to the tab “Datasets”.
    iii. Connect to the record repository in the “Data Portal” tab.
  d. Download the complete data tables (.txt) through the “Table Download” tab.
    i. Request access to record-level hospital inpatient data via Field 41259.
    ii. Request access to record-level primary care data via Field 42038, 42039 and 42040.
    iii. Request access to record-level death register via Field 40023.
3. Obtain the most recent participant withdrawal list for the project.
  a. This list is sent by the UK Biobank to the principal investigator and delegated collaborators and it contains the identifiers of participants who should be excluded in the analyses.

**CRITICAL:** The time required at this step varies depending on the size of dataset that has been approved for the project (please see expected outcomes for examples).

### Download R and required packages

**Timing: 20 min**

4. Install R (R Core Team, 2020) and RStudio (RStudio Team, 2020).
5. Install devtools, ukbpheno, data.table, dplyr, tableone, ggforce, ggplot2, survminer and MatchIt (if not installed) inside an R session.

install.packages("data.table")

install.packages("dplyr")

install.packages("ggplot2")

install.packages("ggforce")

install.packages("tableone")

install.packages("survminer")

install.packages("MatchIt")

install.packages("devtools")

devtools::install_github("niekverw/ukbpheno")

There is no hard requirement on R versions for ukbpheno. Results presented in this protocol were produced running with R version 4.0.3 with RStudio version 1.3.959 in a Unix system.

library(data.table)

library(dplyr)

library(ukbpheno)

library(ggplot2)

library(ggforce)

library(tableone)

library(survminer)

library("MatchIt")

## Key resources table

**Table:** 

REAGENT or RESOURCE	SOURCE	IDENTIFIER
**Software and algorithms**		

R Project for Statistical Computing	R Core Team	RRID: SCR_001905 https://www.r-project.org/
RStudio	RStudio Team	RRID: SCR_000432 http://www.rstudio.com/
ukbpheno	this paper	https://github.com/niekverw/ukbpheno Zenodo: https://doi.org/10.5281/zenodo.6557829
ComplexUpset	The R Foundation	https://cran.r-project.org/package=ComplexUpset
data.table	The R Foundation	https://cran.r-project.org/package=data.table
devtools	The R Foundation	https://cran.r-project.org/package=devtools
dplyr	The R Foundation	https://cran.r-project.org/package=dplyr
fasttime	The R Foundation	https://cran.r-project.org/package=fasttime
ggdendro	The R Foundation	https://cran.r-project.org/package=ggdendro
ggforce	The R Foundation	https:// cran.r-project.org/package=ggforce
ggplot2	The R Foundation	https://cran.r-project.org/package=ggplot2
ggpubr	The R Foundation	https://cran.r-project.org/package=ggpubr
ggrepel	The R Foundation	https://cran.r-project.org/package=ggrepel
glue	The R Foundation	https://cran.r-project.org/package=glue
jsonlite	The R Foundation	https://cran.r-project.org/package=jsonlite
lubridate	The R Foundation	https://cran.r-project.org/package=lubridate
magrittr	The R Foundation	https://cran.r-project.org/package=magrittr
MatchIt	The R Foundation	https://cran.r-project.org/package=MatchIt
matrixStats	The R Foundation	https://cran.r-project.org/package=matrixStats
readxl	The R Foundation	https://cran.r-project.org/package=readxl
RColorBrewer	The R Foundation	https://cran.r-project.org/package=RColorBrewer
stringr	The R Foundation	https://cran.r-project.org/package=stringr
survminer	The R Foundation	https://cran.r-project.org/package=survminer
tableone	The R Foundation	https://cran.r-project.org/package=tableone
tictoc	The R Foundation	https://cran.r-project.org/package=tictoc
XML	The R Foundation	https://cran.r-project.org/package=XML

**Other**		

UK Biobank	UK Biobank	RRID: SCR_012815 http://www.ukbiobank.ac.uk/

## Step-by-step method details

### Build definition table for target health outcome

**Timing: 2–8 h**

Health outcome information from various data sources / data fields within the main dataset is encoded differently. These relationships have been curated and recorded in the data setting file included in the ukbpheno package. For a target phenotype, survey the various data sources/ data fields on the Showcase and determine the definitions for the target phenotype in UK Biobank. An example definition table to define type 2 diabetes is included in the package. This example table can be used as a template for users to define their target health outcomes.

**Figure 3:**
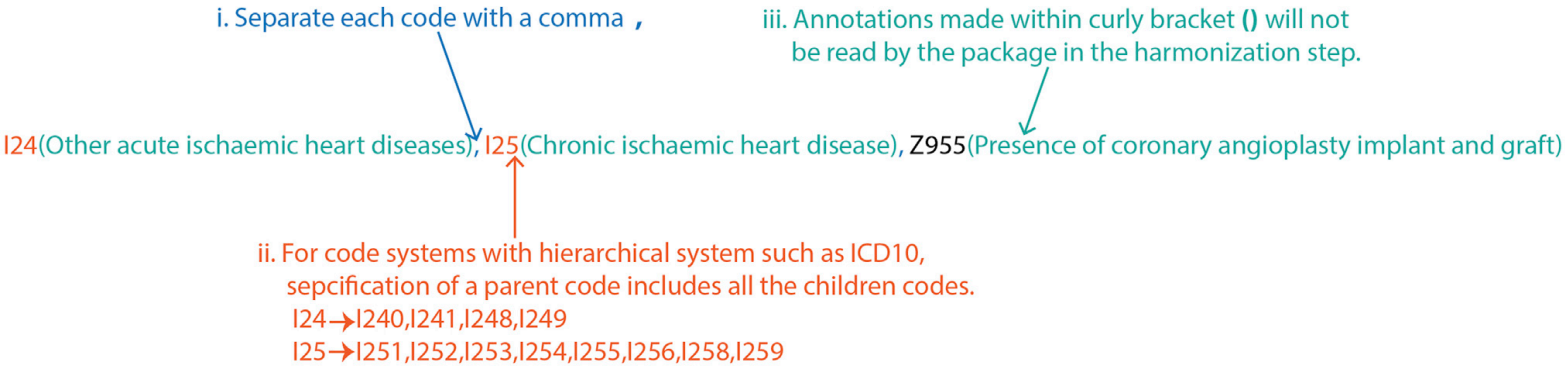
Basic syntax for filling in the definition tables

**Figure 4:**
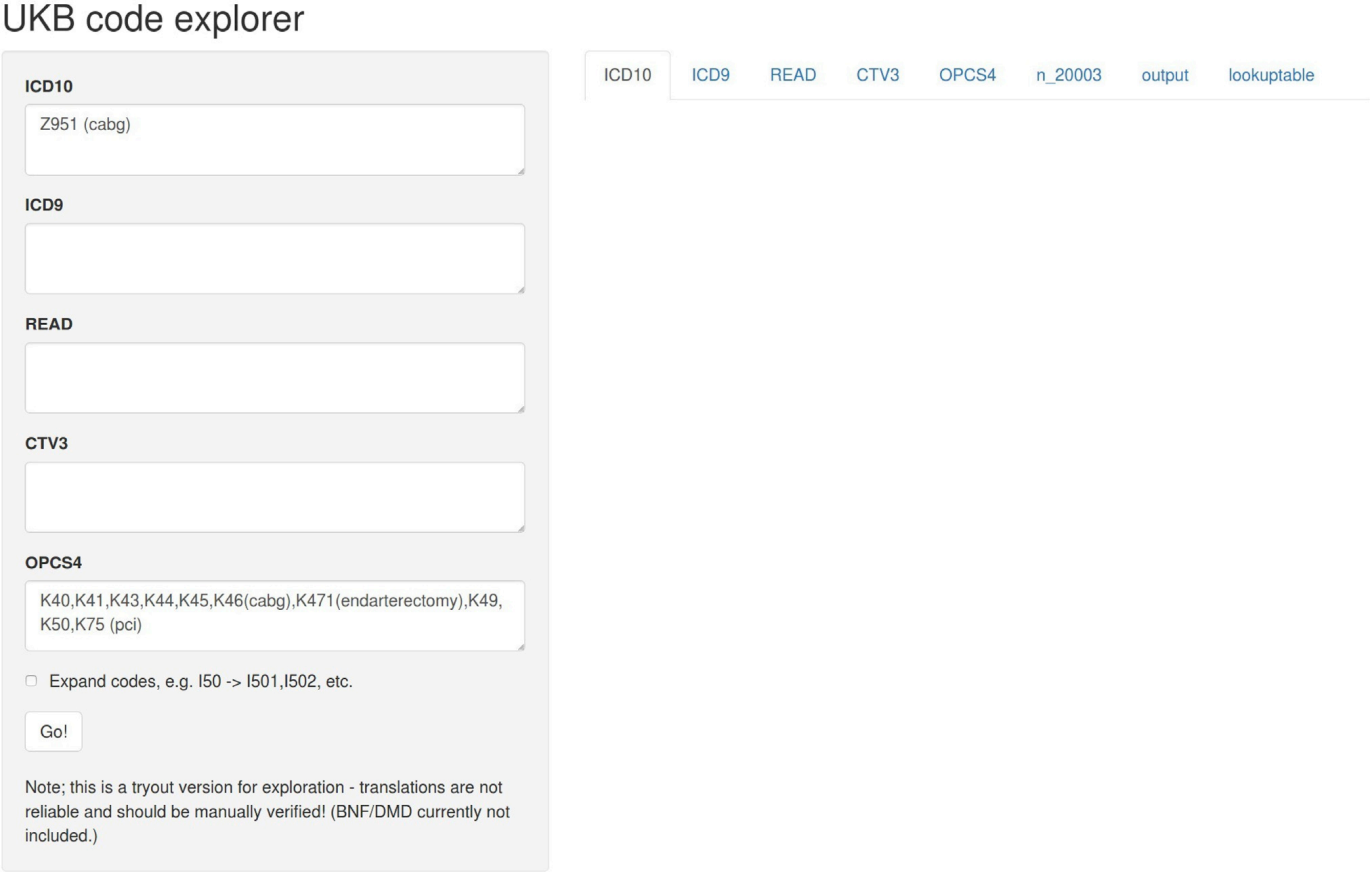
The interface of the shiny app “ukb code explorer”
  b. Fill in fields with conditions in the “TS” (touchscreen) column.
    i. Fill in field number as Showcase followed by the condition e.g., “6177=3(insulin)”
    ii. Table 2 shows the conditions symbols accepted: = (equal), != (not equal), <, <=, >, >=, ≥, ≤

1. Download data setting file (data.settings.tsv) to the project directory from https://github.com/niekverw/ukbpheno/tree/master/inst/extdata/data.settings.tsv.
2. Download definition table template to the project directory from https://github.com/niekverw/ukbpheno/tree/master/inst/extdata/definitions_DmRxT2.tsv.
3. Fill in one phenotype (such as DmT2) per row. The column “TRAIT” contains the unique identifier of each phenotype which is case sensitive.
  a. For each of code systems e.g., diagnosis codes ICD10 or operation codes OPCS4 as well as codes used in the self-report fields, fill in the corresponding codes in the table.
    i. Each code should be separated by a comma.
    ii. For code systems with hierarchical system (refer to data setting file), it is possible to fill in only the parent codes instead of specifying all codes.
    iii. Annotations of the codes can be made using curly bracket “()”.
      Figure 3 illustrates an example for the three rules above.
      ***Optional:*** We included a shiny app to cross-reference codes between systems using the mapping file provided by UK Biobank. (https://github.com/niekverw/ukbpheno/blob/master/inst/util/shiny.lookup_codes.R). Download the code map file (Excel workbook) provided by the UK Biobank (https://biobank.ndph.ox.ac.uk/showcase/refer.cgi?id=592): (1) locate the shiny app script and run the shiny app, and (2) visit the address returned (usually in the form of http://127.0.0.1:xxxx) in a web browser and use the app. A screenshot of the shiny app can be found in Figure 4.

**Table 2:** Syntax accepted by the package to describe conditions

Condition symbol	Meaning
=	Equal to (value)
!=	Not equal to
<	Smaller than
<= OR ≤	Smaller than or equal to
>	Larger than
>= OR ≥	Larger than or equal to
    iii. Add the corresponding age of diagnosis using “[]” following the condition e.g., “4041=1[2976](Gestational diabetes)”
4. It is possible to create a composite phenotype, which involves other phenotypes. Composite phenotypes are constructed using four columns in the definition table (Table 3).
  a. “Study_population” can be used to restrict definition on a subgroup of participants with specific phenotype.
  b. Participants with phenotypes in “Include_definition” will be considered to be a case for the composite phenotype.
  c. Users may use the “Exclude_from_cases” and “Exclude_from_controls” column to exclude participants with certain phenotype(s) from cases and controls respectively.

**Table 3:** Example usage of composite phenotype columns

Exclude_from_cases	Study_population	Exclude_from_controls	Include_definitions
DmT1		RxDm	RxDmOr

In this example usage: cases with records of “DmT1” (type 1 diabetes) are excluded; Controls with records indicating “RxDm” (use of antidiabetic medication) are excluded; participants with records indicating “RxDmOr” (use of oral antidiabetic medication) will be considered as cases for this composite phenotype.

***Note:*** For example, a composite phenotype “diabetes mellitus” may include two phenotypes “type 1 diabetes” and “type 2 diabetes”. Alternatively, for the phenotype “type 2 diabetes” we may want to exclude any cases with also a “type 1 diabetes” diagnosis.

5. The definition table template “definitions_DmRxT2.tsv” contains definitions constructed for the definition of type 2 diabetes in the UK Biobank.
  a. Trait “DmT2”, “DmT1” and “DmG” contain specific codes for diabetes type 2, type 1 and gestational diabetes respectively;
  b. “RxDm” defines the antidiabetic medication which is further divided into “RxDmIns” (Insulin) and “RxDmOr” (oral antidiabetic drugs);
  c. “Dm” captures general codes for diabetes and the remaining definitions are used to differentiate between type 1 and type 2 diabetes within this group.

### Load input files in R

**Timing: 15 min**

Input files required by the package include data files from UK Biobank including the main dataset, the metadata file and optionally data tables from Data Portal; the completed definition table and data setting file.

6. Specify data file paths in R.

# The directory with data files

pheno_dir <-"mydata/ukb99999/"

# Main dataset

fukbtab <- paste(pheno_dir,"ukb99999.tab",sep="")

# Metadata file

fhtml <- paste(pheno_dir,"ukb99999.html",sep="")

# Hospital inpatient data

fhesin <- paste(pheno_dir,"hesin.txt",sep="")

fhesin_diag <- paste(pheno_dir,"hesin_diag.txt",sep="")

fhesin_oper <- paste(pheno_dir,"hesin_oper.txt",sep="")

# GP data

fgp_clinical <- paste(pheno_dir,"gp_clinical.txt",sep="")

fgp_scripts <- paste(pheno_dir,"gp_scripts.txt",sep="")

# Death registry

fdeath_portal <- paste(pheno_dir,"death.txt",sep="")

fdeath_cause_portal <- paste(pheno_dir,"death_cause.txt",sep="")

# Participant withdrawal list

f_withdrawal<-paste(pheno_dir,"w12345_20210809.csv",sep="")

7. Specify files paths for the data setting file, the definition table and code maps which are included in the package (extdata/). Alternatively download the files from code repository of ukbpheno hosted at GitHub.

# Or download the files from

#https://github.com/niekverw/ukbpheno/tree/master/inst/extdata/

extdata_dir<-paste0(system.file("extdata", package="ukbpheno"),"/")

fdefinitions <- paste0(extdata_dir,"definitions_DmRxT2.tsv")

fdata_setting <- paste0(extdata_dir,"data.settings.tsv")

8. Read data setting file. The pre-curated data setting file specifies the characteristics of each data source which are taken into account in the data harmonization process.

dfData.settings <- fread(fdata_setting)

9. Run the “read_definition_table()” function to process the definition table.
  a. The function “read_definition_table()” expands parent codes using the code maps and sort out codes relevant for inclusion and exclusion accordingly.
    i. Code maps include all available codes.
  b. The function will also cross-check codes entered in the definition with the code maps and warn users of any non-matching codes e.g.,
    i. A specific ICD10 code may not exist in the UK Biobank ICD10 code map as this code is not present in the data.
    ii. There may be typos.

dfDefinitions_processed_expanded<-read_defnition_table(fdefinitions,fdata_setting,extdata_dir)

***Optional:*** Alternatively download the code maps from the UK Biobank Showcase or create them manually by extracting all unique codes from your data using “get_all_exisiting_codes()” which generates flat-form (non-hierarchical) code maps. Adjust the data setting file accordingly.

# First input: file path to GP clinical table

# Second input: corresponding column names from the .txt file

# Third input: output file-path

get_all_exsiting_codes(fgp_clinical,c("read_2","read_3"),c("gpclinical.read2.code"," gpclinical.read3.code"))

### Harmonize all data from various sources

**Timing: 15–45 min**

At the harmonization step, we combine all the available data files from various sources and transform them to the format of clinical events to facilitate downstream analyses (Figure 1). For individual level data including the self-report data, cancer registry and optionally death registry, the corresponding fields containing the information on the diagnosis and time of diagnosis are extracted (in the corresponding data types) from the main dataset and converted to the episodes of clinical events. Touchscreen data are processed according to the conditions described in the definition table, if one is provided. The record level data, downloaded from the Data Portal, will be parsed and reorganized by the data source and classification system.

At the end of the harmonization, all clinical events will be returned in the same episode format. In addition, the original data from main dataset and a full list of participants are also returned.

10. Load, process and harmonize all data files using harmonized_ukb_data().
  a. The “allow_missing_fields” flag specifies whether field(s) required on the definition table but missing in the main dataset is allowed and ignored. If this flag is set to “FALSE”, the harmonization step will halt in case of any missing field.
  b. If the participant withdrawal list is provided, records of these individuals will be removed.

***Note:*** The function harmonized_ukb_data() harmonizes all available data (minimally works with only the main dataset and meta-data file). Additionally, the function will check if all fields required on the definition table are present in the main dataset and inform the user if any field is missing.

lst.harmonized.data<-harmonize_ukb_data(f.ukbtab = fukbtab,f.html = fhtml,dfDefinitions=dfDefinitions_processed_expanded,f.gp_clinical = fgp_clinical,f.gp_scripts = fgp_scripts,f.hesin = fhesin,f.hesin_diag = fhesin_diag,f.hesin_oper =fhesin_oper,f.death_portal = fdeath_portal,f.death_cause_portal = fdeath_cause_portal,f.withdrawal_list=f_withdrawal,allow_missing_fields = TRUE)

***Note:*** Time required to harmonize the data is dependent on size of the files. Factors that should be taken into considerations include number of fields approved for the particular project, number of participants included as well as if record-level primary care data are present.

**Figure 5:**
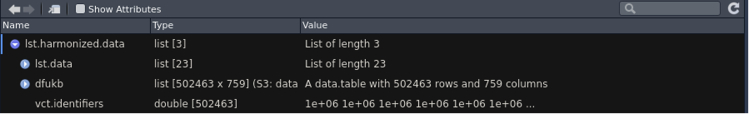
Screenshot of the lst.harmonized.data object

**Figure 6:**
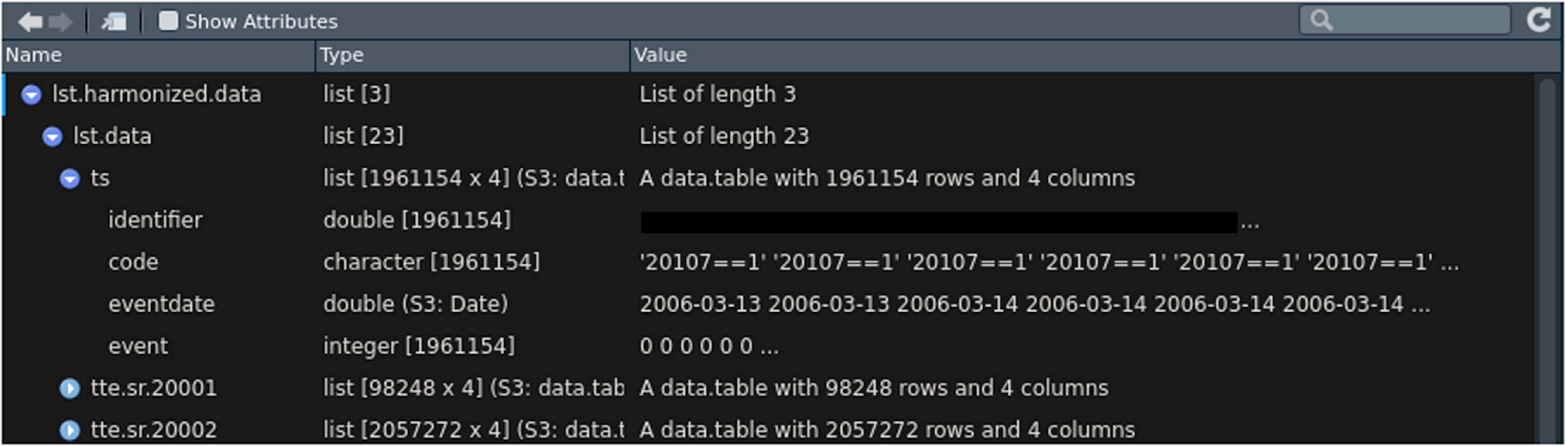
Screenshot of the harmonized records
  b. “dfukb” is a subset of the main dataset and contains only columns necessary for the definition of target phenotypes.
  c. “vct.identifiers” is a vector of identifiers of all participants in the main dataset.

11. Examine the harmonized data which contains 3 objects: “lst.data”, “dfukb” and “vct.identifiers” (Figure 5).
  View(lst.harmonized.data)
  a. “lst.data” contains data from all sources in same episode format documenting “identifier”, “code”,”eventdate” and an “event” column.
    i. Diagnosis codes without associated actual event date will have date of visit to assessment center (such as self-report diabetes) in the “eventdate” column and “0” in the “event” indicating that the date does not reflect a true event (Figure 6).
      View(lst.harmonized.data$lst.data)

### Generate phenotype and explore the data

**Timing: 2 h**

12. To define case/control status of the participants, we need the phenotype (diabetes) definition, the harmonized data tables, the data settings and the individuals to be included (either specified by a vector of participant identifiers or a data-frame containing identifier in the first column and reference dates in the second column).

# 1) definition of the target trait “Type 2 diabetes”

trait<-"DmRxT2"

# 2) harmonized data table - lst.harmonized.data

# 3) data setting data-frame - dfData.settings

# 4) individuals specified via df_reference_date

# Here the dates of baseline visit (f.53.0.0) are taken as reference

df_reference_dt_v0<-

lst.harmonized.data$dfukb[,c("identifier","f.53.0.0")]

13. Use “get_cases_controls()” function to obtain the case/control status. The function returns a list of three data.table objects: “df.casecontrol”, “all_event_dt.Include_in_cases” and “all_event_dt.Include_in_cases.summary” (Figure 7).
  lst.DmRxT2.case_control <- get_cases_controls(definitions=dfDefinitions_processed_expanded %>% filter(TRAIT==trait), lst.harmonized.data$lst.data,dfData.settings, df_reference_date=df_reference_dt_v0)
  View(lst.DmRxT2.case_control)
  a. “df.casecontrol” is a data.table object of 16 columns providing summary of the diagnosis per participant (Table 4). Included case/control is marked with 2/1 respectively while excluded case/control will be marked with -2/-1 in this table.

**Table 4:** Column description of the case-control summary table

Column name	Information
identifier	Unique identifier of the participant
reference_date	The reference dates supplied by user
count	Number of episodes/events for that participant related to the target phenotype (diagnosis)
sum.epidur	Total number of days hospitalized due to the diagnosis according to secondary care data
median.epidur	The median days of hospitalization according to secondary care data
max.epidur	The number of days from the longest hospital stay due to the diagnosis.
survival_days	Days of survival from the reference date if the participant had died of the diagnosis as evidenced from the death registry
Death_primary	Indicates if the participant has died with the diagnosis as primary cause
Death_any	Indicates if the participant has died with the diagnosis as either primary or secondary cause
Hx_days	Duration of diagnosis in days counting at the reference date
Fu_days	Follow-up time until the participant has the diagnosis counting from the reference date
Hx	Indicate if the participant has the diagnosis before the reference date (prevalent case)
Fu	Indicate if the participant has the diagnosis after the reference date (incident case)
Ref	Indicate if the diagnosis was made close to the reference date with a window (default: 0 day)
first_diagnosis_days	Difference between first occurrence and reference date (including both Hx and Fu)
Any	Indicate if the participant has a diagnosis (including both Hx and Fu)
  b. “all_event_dt.Include_in_cases” is data.table object including all event episodes supporting the diagnosis for the cases included (Table 5).

**Table 5:** Column description of the case event table

Column name	Information
.id	Source of this event
identifier	Unique identifier of the participant
code	Event code
eventdate	Event date
event	Indicate if this episode contains a true event date (event date from linked data or self-report operation=1, self-report event date except for operations =2, not a true event date=0)
epidur	Days hospitalized in this episode documented in secondary care data
classification	Refers to the classification system of the code
  c. “all_event_dt.Include_in_cases.summary” is a data.table object with the same format with “df.casecontrol” but includes only cases (both included and excluded case).

**Figure 7:**
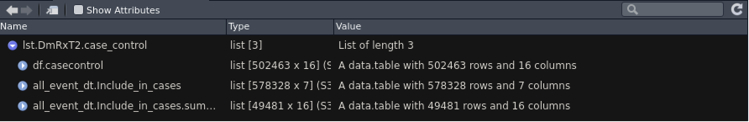
Screenshot of the result obtained from the get_cases_controls() function
14. Generate timeline plot to check the relative contribution by various data sources over time (Figure 8). Events with known event date will be included.

**Figure 8:**
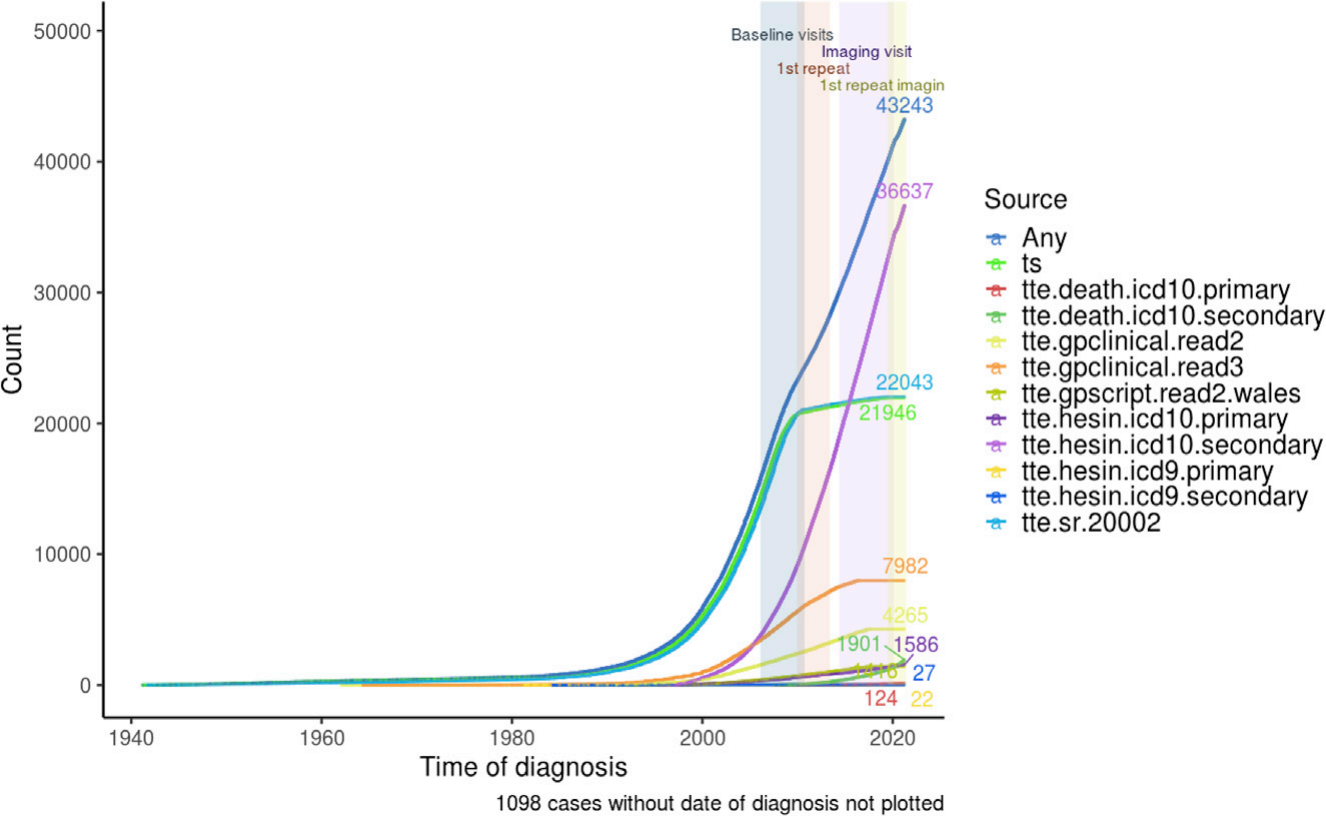
Disease timeline of type 2 diabetes by different data sources

DmRxT2_timeline<-plot_disease_timeline_by_source(definition=dfDefinitions_processed_expanded%>%filter(TRAIT==trait),lst.harmonized.data$lst.data,dfData.settings, df_reference_dt_v0$identifiers)

DmRxT2_timeline

**Figure 9:**
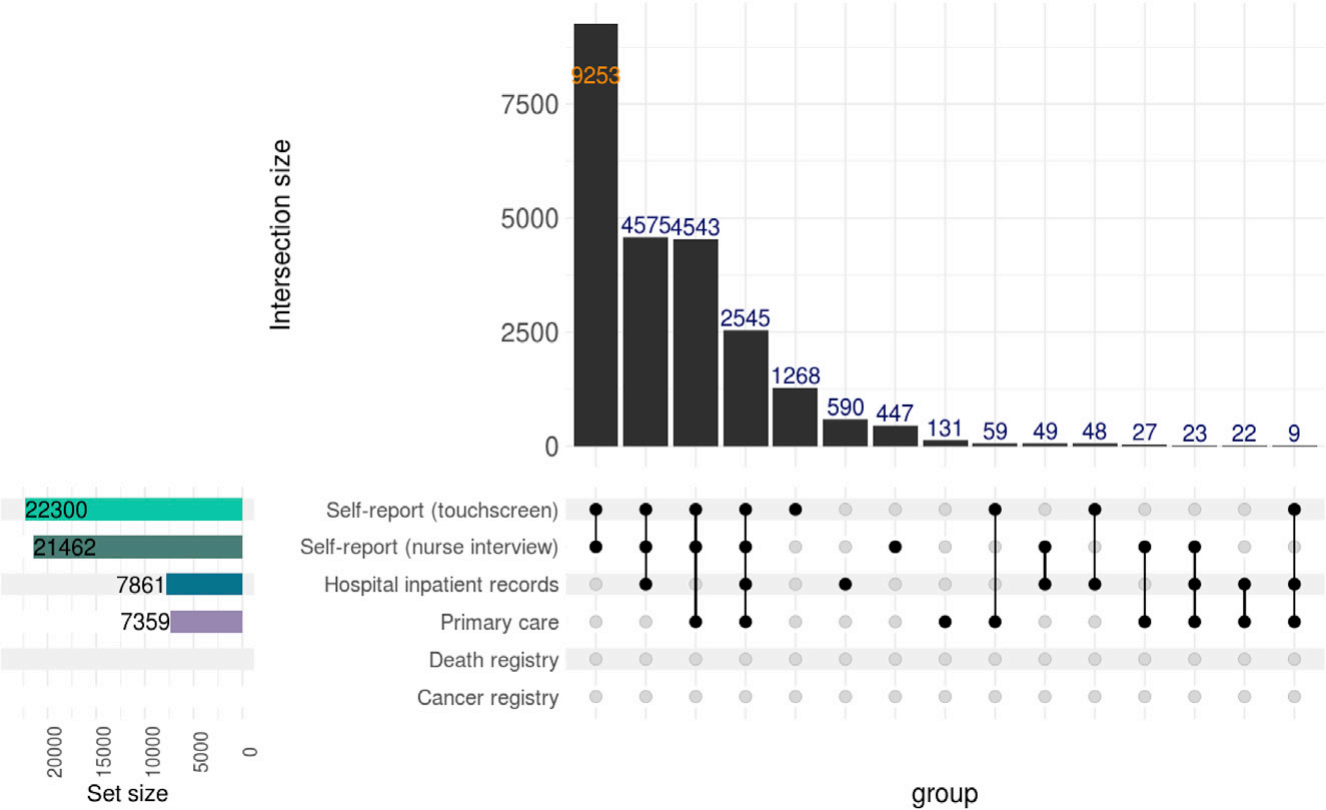
UpSet plot of type 2 diabetes at baseline showing the overlaps between different data sources

15. Use “make_upsetplot()” to examine the overlaps between the data sources at baseline to gain insight on their relationships (Figure 9).

upset_plot<-make_upsetplot(definition=dfDefinitions_processed_expanded%>%filter(TRAIT==trait),lst.harmonized.data.gp$lst.data,dfData.settings,df.reference.dates = df_reference_dt_v0)

upset_plot

**Figure 10:**
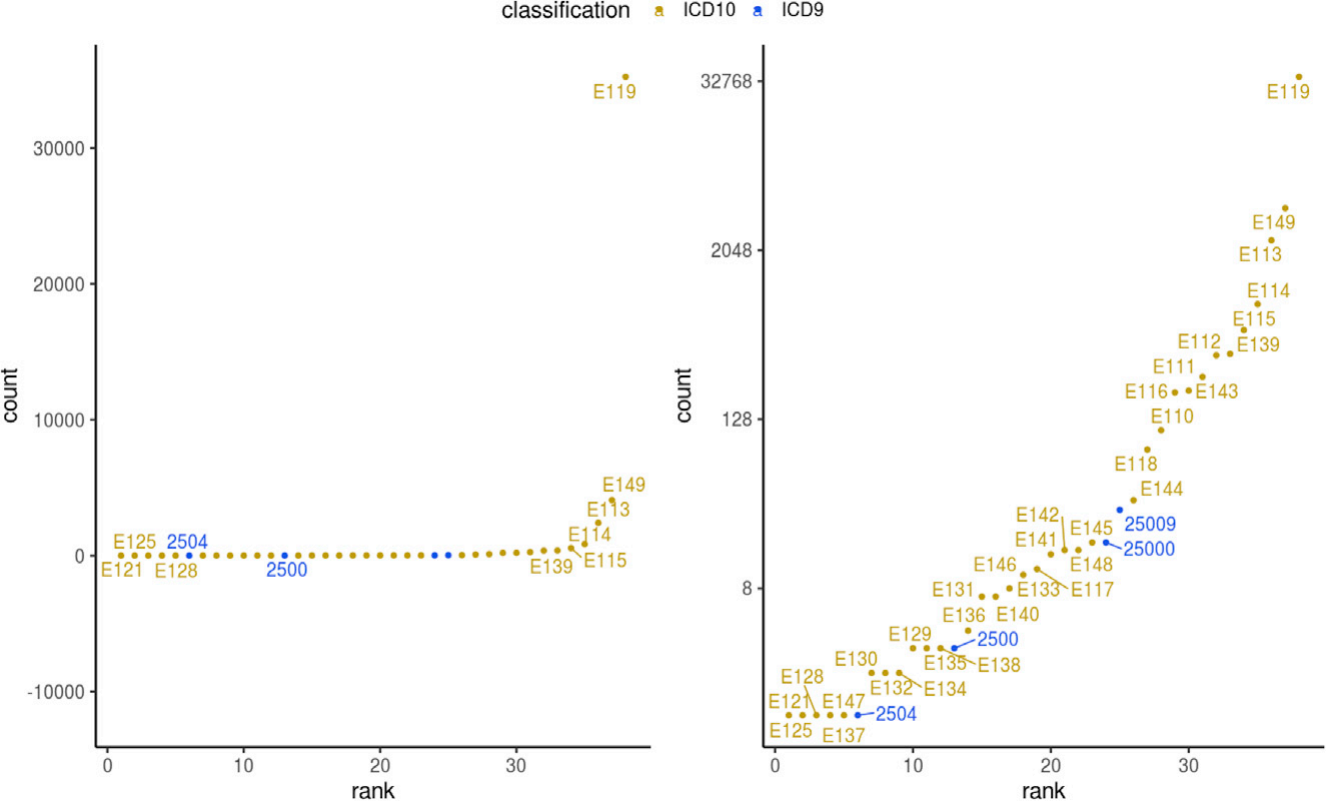
Frequency plots of type 2 diabetes diagnosis codes from secondary care Left: y-axis in linear scale; Right: y-axis in logarithmic scale.

16. Generate summary descriptions on the events with “get_stats_for_events”. For example, generation of a frequency plot of codes among all events from secondary care may help verify or refine the definition (Figure 10).

# Extract all hospital admission records

all_DmRxT2_evnt<-lst.DmRxT2.case_control$all_event_dt.Include_in_cases

DmRxT2_hesin_rec<-all_DmRxT2_evnt[grepl ("hesin",all_DmRxT2_evnt$.id)]

# Get some descriptive statistics on the records on a code level

hesin_stats<-get_stats_for_events(DmRxT2_hesin_rec)

hesin_stats$stats.codes.summary.phesin_stats$stats.codes.summary.p

**Figure 11:**
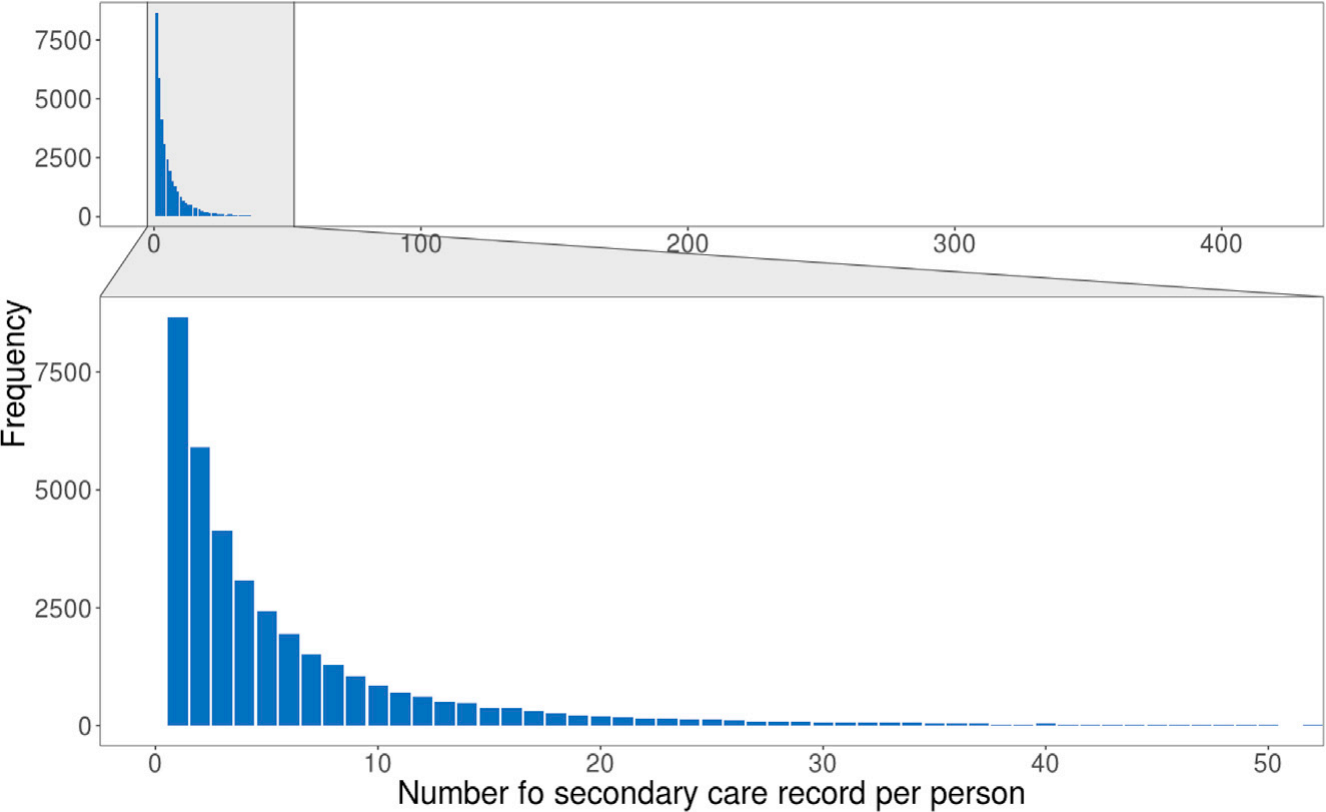
Barplot of type 2 diabetes diagnosis code count from secondary care per individual

17. Explore secondary care code count by individual (Figure 11).

# Get some summary statistics on the records on individual level

DmRxT2_rec_cnt<-DmRxT2_hesin_rec[,.(count=.N),by=c("identifier")]

max(DmRxT2_rec_cnt$count)

median(DmRxT2_rec_cnt$count)

mean(DmRxT2_rec_cnt$count)

quantile(DmRxT2_rec_cnt$count)

# Visualize count with barplot with a zoom-in on count between 0-50

ggplot2::ggplot(DmRxT2_rec_cnt, ggplot2::aes(x=count)) +

 ggplot2::geom_bar(fill="#0073C2FF") + ggplot2::xlab("Number fo secondary care record per person") +

 ggplot2::ylab("Frequency") + #theme with white background

 ggplot2::theme_bw() + ggplot2::theme(text = ggplot2::element_text(size=22),panel.grid.minor =ggplot2::element_blank(),panel.grid.major =ggplot2::element_blank()) + ggforce::facet_zoom(xlim = c(0, 50))

**Figure 12:**
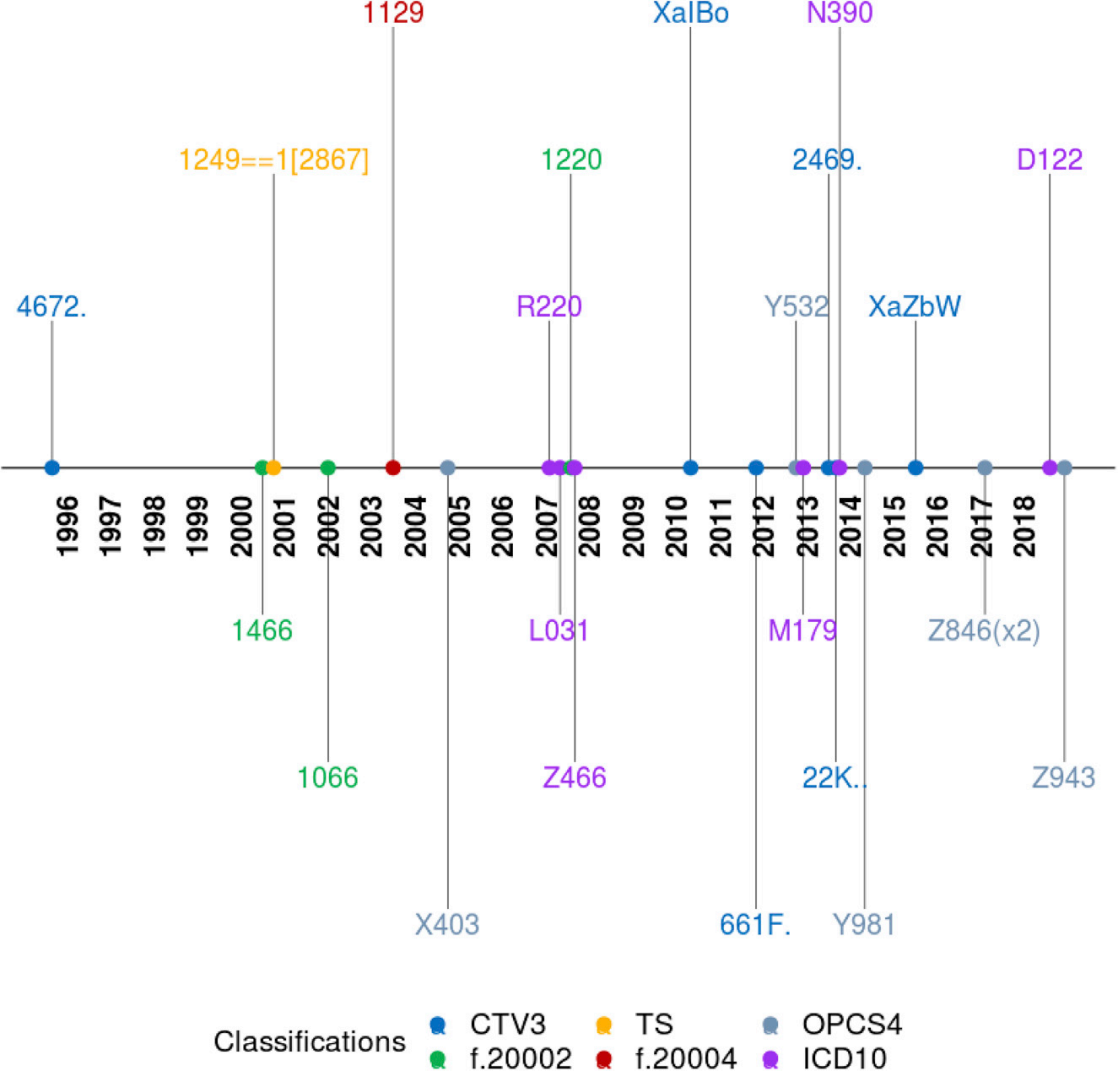
Diagnosis timeline of a hypothetical participant

18. Generate a timeline of the codes contributing to diagnosis for a particular individual (please replace the identifier if copied from the cell below) (Figure 12).

# Plot individual time line

plot_individual_timeline(df.data.settings = dfData.settings,lst.data=lst.harmonized.data$lst.data,ind_identifier = 9999999)

To make the definition of the type 2 diabetes more precise, we may screen and exclude individuals with evidence of other types of diabetes as well as the use of metformin not due to diabetes.

19. First identify participants with specific diabetes codes (gestational diabetes, type 1 and type 2 diabetes) as well as general diabetes code.

# Identify individuals with specific DmT2 codes

lst.DmT2.case_control<-get_cases_controls(definitions=dfDefinitions_processed_expanded %>% filter(TRAIT=="DmT2"), lst.harmonized.data$lst.data,dfData.settings, df_reference_date=df_reference_dt_v0)

# Identify individuals with specific DmT1 codes

lst.DmT1.case_control<-get_cases_controls(definitions=dfDefinitions_processed_expanded %>% filter(TRAIT=="DmT1"), lst.harmonized.data$lst.data,dfData.settings, df_reference_date=df_reference_dt_v0)

# Identify individuals with DmG

lst.DmG.case_control <- get_cases_controls(definitions=dfDefinitions_processed_expanded %>% filter(TRAIT=="DmG"), lst.harmonized.data$lst.data,dfData.settings, df_reference_date=df_reference_dt_v0)

# Identify individuals with general diabetes diagnosis codes excl. medication

lst.Dm.case_control <- get_cases_controls(definitions=dfDefinitions_processed_expanded %>% filter(TRAIT=="Dm"), lst.harmonized.data$lst.data,dfData.settings, df_reference_date=df_reference_dt_v0)

20. Identify use of different anti-diabetic medications. Find individuals on metformin likely due to diseases other than diabetes by cross checking with the list of individuals with diabetes diagnoses.

# Identify individuals with metformin use

lst.RxMet.case_control <- get_cases_controls(definitions=dfDefinitions_processed_expanded %>% filter(TRAIT=="RxMet"), lst.harmonized.data$lst.data,dfData.settings, df_reference_date=df_reference_dt_v0)

#Identify use of insulin/oral diabetic med. excl. metformin

lst.RxDmNoMet.case_control <- get_cases_controls(definitions=dfDefinitions_processed_expanded %>% filter(TRAIT=="RxDmNoMet"), lst.harmonized.data$lst.data,dfData.settings, df_reference_date=df_reference_dt_v0)

#Identify individuals that are on metformin but no diabetes codes

#nor medication other than metformin

RxMet_DmUnlikely<-setdiff(lst.RxMet.case_control$df.casecontrol[Hx==2]$identifier,union(lst.Dm.case_control$df.casecontrol[Hx==2]$identifier,lst.RxDmNoMet.case_control$df.casecontrol[Hx==2]$identifier))

21. Cross-examine various diagnoses. For example we want to get individuals with young onset diabetes but did not have records supporting a diagnosis of non-type 2 diabetes. Namely these individuals did not have evidence of type 1 diabetes nor gestational diabetes.
  a. We identify these individuals via set operations of the relevant diagnoses.
  b. Inspect the records of these individuals for evidence of type 2 diabetes.

# Identify individuals with self-report insulin <12 months post-diagnosis

lst.RxDmInsFirstYear.case_control<-get_cases_controls(definitions=dfDefinitions_processed_expanded %>% filter(TRAIT=="RxDmInsFirstYear"), lst.harmonized.data$lst.data,dfData.settings, df_reference_date=df_reference_dt_v0)

# Identify young onset self reported diabetes (European origin)

lst.SrDmYEw.case_control <- get_cases_controls(definitions=dfDefinitions_processed_expanded %>% filter(TRAIT=="SrDmYEw"), lst.harmonized.data$lst.data,dfData.settings, df_reference_date=df_reference_dt_v0)

# identify young onset self reported diabetes (Caribbean African origin)

lst.SrDmYSaCa.case_control <- get_cases_controls(definitions=dfDefinitions_processed_expanded %>% filter(TRAIT=="SrDmYSaCa"), lst.harmonized.data$lst.data,dfData.settings, df_reference_date=df_reference_dt_v0)

# Individuals of young onset diabetes

ind_young_onset<- union(lst.SrDmYSaCa.case_control$df.casecontrol[Any==2]$identifier,lst.SrDmYEw.case_control$df.casecontrol[Any==2]$identifier)

# Individuals with evidence of other types of diabetes reported

ind_RxInsFirstYear_DmT1_DmG<- union(union(lst.RxDmInsFirstYear.case_control$df.casecontrol[Any==2]$identifier,lst.DmT1.case_control$df.casecontrol[Hx==2]$identifier),lst.DmG.case_control$df.casecontrol[Hx==2]$identifier)

# Young onset but no DM type 1/ gestational diabetes specific codes nor self report of insulin within first year of diagnosis

inds_young_onset_possible_DmT2 <-setdiff(ind_young_onset,ind_RxInsFirstYear_DmT1_DmG)

# Check the records of these individuals

lst.DmRxT2.case_control$all_event_dt.Include_in_cases[identifier %in% inds_young_onset_probable_DmT2]

### Generate phenotypes in batch

**Timing: 15–30 min**

This session demonstrates how to generate multiple phenotypes and make a clinical characteristics table with these phenotypes, stratified by type 2 diabetes status. An example definition table with the selected cardiometabolic diseases, family history of these diseases and diabetes medication usage is provided in the package. We additionally extract demographic information namely age and sex as well as biomarkers BMI, blood glucose, glycated hemoglobin and self-report insulin use within one year of diabetes diagnosis from the main dataset.

22. Read and process the definition file.

# Read the definitions table

fdefinitions <- paste0(extdata_dir,"definitions_cardiometabolic_traits.tsv")

dfDefinitions_processed_expanded<-read_defnition_table(fdefinitions,fdata_setting, extdata_dir)

23. Extract only the required fields from the main dataset using read_ukb_tabdata().
  a. The metadata provides information such as data type of these fields.
  b. Extract age at assessment center visit (Field 21003), sex (Field 31), body mass index (Field 21001), glycated hemoglobin level (Field 30750), glucose level (Field 30740), self-report insulin use within the first year of diabetes diagnosis (Field 2986), UK Biobank assessment center visited (Field 54) and Date of attending assessment center (Field 53).

# Extract clinical variables from the main dataset using read_ukb_tabdata()

# We need the metadata (.html) file for read_ukb_tabdata()

dfhtml <- read_ukb_metadata(fhtml)

# Rename the identifier column in the metadata

dfhtml[which(dfhtml$field.tab=="f.eid"),]$field.tab<-"identifier"

# Age at assessment center visit, sex, BMI, HbA1c, glucose,insulin within 1 year of diagnosis,UK Biobank assessment center location, date of visit

baseline_fields<-c(21003,31,21001,30750,30740,2986,54,53)

# Extract these variables from main dataset

dfukb_baseline <- read_ukb_tabdata(fukbtab,dfhtml,fields_to_keep = baseline_fields)

gc()

24. Generate the phenotypes for atrial fibrillation, coronary artery disease, type 2 diabetes, hypertrophic cardiomyopathy, heart failure, hypertension and hyperlipidemia with a loop and merge the phenotype information into one table “dfukb_baseline_pheno”.

# The target disease traits we will generate in batch

diseases<-c("Af","Cad","DmT2","Hcm","Hf","HtRx","HyperLipRx")

# Make an output folder to store the result

out_folder<-paste0(pheno_dir,"output/")

if(!dir.exists(file.path(out_folder))){

dir.create(file.path(out_folder))

}

df_withdrawal<-fread(f_withdrawal)

# remove withdrawn participants

dfukb_baseline_pheno<-dfukb_baseline[! identifier %in% df_withdrawal$V1]

# Loop through the traits, including family history of related diseases and the diabetes medication use

for (disease in c(diseases,"HxDm","HxHrt","HxHt","RxDmOr","RxDmIns")){

print(disease)

lst.case_control <- get_cases_controls(definitions=dfDefinitions_processed_expanded %>% filter(TRAIT==disease), lst.harmonized.data$lst.data,dfData.settings, df_reference_date=df_reference_dt_v0)

 # Add the trait to the column names

colnames(lst.case_control$df.casecontrol) <- paste(disease,"0",colnames(lst.case_control$df.casecontrol), sep = "_")

 # Except for participant identifier

names(lst.case_control$df.casecontrol)[names(lst.case_control$df.casecontrol) == paste(disease,"0","identifier", sep = "_")]<-"identifier"

# Merge these columns with dfukb_baseline_pheno

dfukb_baseline_pheno<-merge(dfukb_baseline_pheno,lst.case_control$df.casecontrol,by="identifier",all.x = TRUE,all.y = FALSE)

}

### Report clinical characteristics at baseline

**Timing: 10 min**

In the following example analyses, we investigate the characteristics of participants with type 2 diabetes specific codes. We exclude the cases with type 1 diabetes diagnosis codes and we exclude any controls with non-specific diabetes codes (Table 6).

25. Select variables to be reported in the clinical characteristics table. Rename the variables in the table to improve readability. Create the clinical characteristics table stratified by type 2 diabetes. Write the clinical characteristic table to a file.

# Keep only the variables needed for the table

dfukb_baseline_pheno_fortable1<-dfukb_baseline_pheno[,c('identifier',"DmT2_0_Hx","f.21003.0.0","f.21001.0.0","f.30740.0.0","f.30750.0.0","DmT2_0_first_diagnosis_days","f.31.0.0","HxDm_0_Any","HxHrt_0_Any","HxHt_0_Any","HtRx_0_Hx","HyperLipRx_0_Hx","Af_0_Hx","Hcm_0_Hx","Hf_0_Hx","RxDmOr_0_Hx","RxDmIns_0_Hx","f.2986.0.0"),with=FALSE]

# Negative first diagnosis day indicates history while positive indicates follow-up cases

dfukb_baseline_pheno_fortable1$DmT2_0_first_diagnosis_years<-(-1∗dfukb_baseline_pheno_fortable1$DmT2_0_first_diagnosis_days)/365.25

# Rename for readability

colnames(dfukb_baseline_pheno_fortable1)<-c("identifier","Type 2 diabetes","Age","BMI","Glucose","HbA1c","Days since type 2 diabetes diagnosis","Sex","Family history of diabetes","Family history of heart disease","Family history of hypertension","Hypertension","Hyperlipidemia","Atrial fibrillation","Hypertrophic cardiomyopathy","Heart failure","Oral diabetes medication","Insulin","Insulin within 1 year of diagnosis","Years since type 2 diabetes diagnosis")

# Below the parameters for CreateTableOne

# The full variable list

vars<-c("Age","BMI","Glucose","HbA1c","Years since type 2 diabetes diagnosis","Sex","Family history of diabetes","Family history of heart disease","Family history of hypertension","Hypertension","Hyperlipidemia","Atrial fibrillation","Hypertrophic cardiomyopathy","Heart failure","Oral diabetes medication","Insulin","Insulin within 1 year of diagnosis")

# The categorical variables on the clinical characteristics table

factorVars<-setdiff(vars,c("Age","BMI","Glucose","HbA1c","Years since type 2 diabetes diagnosis"))

# Create the clinical characteristic table stratified by type 2 diabetes

tableOne <- CreateTableOne(vars = vars, strata = "Type 2 diabetes", data = dfukb_baseline_pheno_fortable1, factorVars = factorVars)

hist(dfukb_baseline_pheno_fortable1$`Years since type 2 diabetes diagnosis`)

tableOne

tab1Mat <- print(tableOne, quote = FALSE, noSpaces = TRUE, printToggle = FALSE,nonnormal =c("Glucose","HbA1c","Years since type 2 diabetes diagnosis"))

# Save the table to a CSV file

write.csv(tab1Mat, file =paste0(out_folder,"BaselineTable.csv"))

**Table 6:** Inclusion and exclusion criteria for the phenotype type 2 diabetes

Exclude_from_cases	Study_population	Exclude_from_controls	Include_definitions
DmT1		DmRx	

### Survival analysis on heart failure stratified by type 2 diabetes

**Timing: 5 min**

26. With time-to-event data as well as the censoring dates for different data sources for different regions, compute the observed time for each participant.
  a. The start time is the date when the participant visited the assessment center;
  b. Observed time is up to date of event or earliest among date of death and censoring date of hospital inpatient records (last follow up).

# Get death dates from data

deathdt<-unique(lst.harmonized.data$lst.data$tte.death.icd10.primary[,.(identifier,eventdate)])

# Rename the column and merge

colnames(deathdt)<-c("identifier","deathdt")

dfukb_baseline_pheno<-merge(dfukb_baseline_pheno,deathdt,by="identifier",all.x=TRUE,all.y = FALSE)

# HESIN censoring date are different by regions

# Use the UK Biobank assessment center location attended by the participants

england<-c("10003","11001","11002","11007","11008","11009","11010","11011","11012","11013","11014","11016","11017","11018","11019","11020","11021")

scotland<-c("11004","11005")

wales<-c("11003","11022", "11006","11023")

# Corresponding censoring dates

dfukb_baseline_pheno[dfukb_baseline_pheno$f.54.0.0 %in% england,"censordateHES"]<-as.Date("2021-03-31")

dfukb_baseline_pheno[dfukb_baseline_pheno$f.54.0.0 %in% scotland,"censordateHES"]<-as.Date("2021-03-31")

dfukb_baseline_pheno[dfukb_baseline_pheno$f.54.0.0 %in% wales,"censordateHES"]<-as.Date("2018-02-28")

# Time-to-event/observed time is determined at earliest of date of event, date of death and censoring date of HESIN data (last follow up)

# This is already calculated for those who have events

range(dfukb_baseline_pheno[Hf_0_Fu==2,Hf_0_Fu_days])

# non-event but died before HESIN censoring date

dfukb_baseline_pheno[Hf_0_Fu==1 & !is.na(deathdt) & deathdt-censordateHES<=0,Hf_0_Fu_days:=deathdt-as.Date(f.53.0.0)]

# People censored at last fu

# non-event but died after censoring date (HESIN),

dfukb_baseline_pheno[Hf_0_Fu==1 &!is.na(deathdt)& deathdt-censordateHES>0,Hf_0_Fu_days:=censordateHES-as.Date(f.53.0.0)]

# non-event and alive by censoring date

dfukb_baseline_pheno[Hf_0_Fu==1 &is.na(deathdt),Hf_0_Fu_days:=censordateHES-as.Date(f.53.0.0)]

27. Create the survival object and Kaplan-Meier plot for new onset heart failure stratified by type 2 diabetes status at baseline.

#Estimate risk of new onset heart failure by presence/absence of type 2 diabetes at baseline

fit<-survival::survfit(survival::Surv(Hf_0_Fu_days/365.25,Hf_0_Fu) ∼ DmT2_0_Hx, data = dfukb_baseline_pheno[DmT2_0_Hx>0])

# summary(fit)

# Make Kaplan-Meier plot

ggsurvplot(fit, data = dfukb_baseline_pheno[DmT2_0_Hx>0], size = 0.8,

 break.time.by=2,

 xlab = "Follow up (years)",

 censor.size=2,

 palette = c("#072A6C", "#FF8400"),

 conf.int = TRUE, # Add confidence interval

 pval = TRUE, # Add p-value

 risk.table = TRUE, # Add risk table

 risk.table.col = "strata", # Risk table color by groups

 legend.labs = c("No type 2 diabetes at baseline","Type 2 diabetes at baseline"),

 risk.table.height = 0.2)

### Case-control matching with MatchIt

**Timing: 5 min**

Sometimes it may be desirable to match cases and controls by characteristics such as age and sex in certain studies. Here we demonstrate how to further process phenotypes created in ukbpheno to generate matched case-control pairs. We utilize an R package MatchIt for the matching task.

28. To match type 2 diabetes case to control by age, sex and body mass index. We extract those variables and remove individuals with missing values in either the target phenotype or any covariates.

########################################

# 1:2 case control matching with MatchIt

########################################

#library(“MatchIt”)

# Remove individuals with either missing or excluded phenotype for target phenotype (type 2 diabetes at baseline)

df_to_matchit<-dfukb_baseline_pheno[!is.na(DmT2_0_Hx) & DmT2_0_Hx>0]

# Pick three covariates age at assessment center visit, sex and BMI for matching

df_to_matchit<-na.omit(df_to_matchit[,.(identifier,DmT2_0_Hx,f.21003.0.0,f.31.0.0,f.21001.0.0)])

29. Format the coding of the phenotype and name the rows by participant identifier in preparation for the matchit() function. Run the matchit() function to match 2 controls to each case. Examine the result.

# Format the data for the matchit function

# Control/case: 1/2 to 0/1

df_to_matchit$DmT2_0_Hx<-df_to_matchit$DmT2_0_Hx-1

# Name the rows

rownames(df_to_matchit)<-df_to_matchit$identifier

colnames(df_to_matchit)<-c("identifier","Type 2 diabetes","Age","Sex","BMI")

# Run matchit

m.dm2<-matchit(`Type 2 diabetes`∼Age + Sex+BMI,data=df_to_matchit,ratio=2)

#Check result

summary(m.dm2)

# Each row in the match.matrix shows identifier of one case with 2 matched controls

m.dm2$match.matrix

## Expected outcomes

### Harmonization of UK Biobank data

After the harmonization step, the user should have in the R workspace the data from both Data Portal (record level data) as well as a subset of the main dataset relevant for the target phenotypes (as specified in the definition table). Users can perform further analyses in R as they see fit (see reporting of clinical characteristics, survival analysis and case-control matching sections).

Using our machine with 64 GB RAM (DDR4 2667 MHz) and 10-core processor (Intel Skylake) under an Ubuntu 16 system, it took 12 min to load and process the main dataset and all record level files. Of which the most time-consuming step was loading the main dataset (six minutes with file size 35.5 GB) followed by the primary care data (three and half minutes with total file size 8.5 GB). The harmonization step was additionally tested on an Ubuntu 16 machine with 16 GB RAM (DDR4 2667 MHz) and 6-core processor (AMD Ryzen 5). It took approximately 45 minutes to complete on this machine.

### Ascertainment of diabetes status

The “get_cases_controls()” function from ukbpheno determines the case/control status for a target phenotype following the inclusion/exclusion criteria outlined in the definition table. Users can extract prevalent as well as incident cases with a specific reference time point. Results presented in this protocol are produced using data downloaded in June 2021, with primary care data for around 45% of the UK Biobank cohort available and data censoring dates shown in Table 7 (UK Biobank, 2022b).

**Table 7:** Censoring dates of different data sources

Data (provider)	Censoring date
Primary care - England (TPP)	31May2016
Primary care - England (Vision)	31May2017
Primary care - Scotland	31Mar2017
Primary care - Wales	31Aug2017
Secondary care - England	31Mar2021
Secondary care - Scotland	31Mar2021
Secondary care - Wales	28Feb2018
Cancer - England/Wales	31Jul2019
Cancer - Scotland	31Oct2015
Death - England/Wales	28Feb2021
Death - Scotland	28Feb2021

The censoring dates for the current release can be found in Showcase (https://biobank.ndph.ox.ac.uk/showcase/exinfo.cgi?src=Data_providers_and_dates).

Following the example definition of “DmRxT2”, users should expect a prevalence of 4.7% at the baseline visit, an estimate close to (Eastwood et al., 2016). Additionally, it can be observed in the disease timeline (Figure 8) as well as the UpSet plot (Figure 9) that the majority of prevalent cases during baseline visit periods were attributed to self-report sources rather than secondary care (hesin) and hence use of secondary care alone for ascertainment would miss out a lot of cases, as reported by Eastwood and colleagues.

Examining the diabetes diagnoses captured in secondary care system, user should be able to obtain similar frequency plots as shown in Figure 10. Counts of diagnosis code per individual have a highly skewed distribution with median of 3 records from the secondary care (Figure 11).

With the plot_individual_timeline() function, user should expect visualization of diagnosis codes of a selected participant over time. Figure 12 shows such a timeline of a hypothetical participant.

Lastly in the cross-examinations with other types of diabetes and medication, users should expect similar numbers as reported in the prevalence algorithms by Eastwood and colleagues (Eastwood et al., 2016). Users could verify these conditions by inspecting the records in the harmonized data or further investigate with other information (such as the biomarker / genetics). For instance, users should be able to observe that around 300 individuals with young onset diabetes received an E119 diagnosis after their baseline visits.

### Phenotype generation in batch

In this part users should be able to generate the cardiometabolic phenotypes namely, atrial fibrillation or flutter, coronary artery disease, hypertrophic cardiomyopathy, heart failure, type 2 diabetes, hypertension and hyperlipidemia. Users should also obtain the related phenotypes including family history of diabetes, family history of heart disease, family history of hypertension and the use of diabetes medications. Using our machine with 64 GB RAM (DDR4 2667 MHz) and 10-core processor (Skylake), it took three minutes to process all 12 phenotypes.

### Report clinical characteristics at baseline and survival analysis

In this part users should obtain a clinical characteristics table stratified by type 2 diabetes status (Table 8). The diagnosis was ascertained with only type 2 diabetes specific diagnosis codes; potential cases with type 1 diabetes specific diagnosis codes (at any time point) were excluded; potential controls with any diabetes diagnosis codes or medications were also excluded. This resulted in four stratified groups in the table – “Case inclusion”, “Case exclusion”, “Control inclusion” and “Control exclusion”. It can be observed that the group of “Case exclusion” had the highest blood glucose and glycated hemoglobin. The proportion of “Case exclusion” (with specific type 1 diabetes diagnosis codes) on insulin was also the highest with the highest percentage of starting insulin within the first year of diagnosis. Higher blood glucose and glycated hemoglobin as well as proportion of diabetes medication can be observed in the group of “Control exclusion” (with non-specific diabetes diagnosis codes - evidence of diabetes but no type 1 / type 2 specific records at baseline) than the “Control inclusion” group. The non-zero percentage of use of diabetes medication (oral diabetes medication / insulin) at baseline observed in the “Control inclusion” group was likely individuals with pre-diabetes; while the negative values of “Years since type 2 diabetes diagnosis” observed were due to the individuals who received diabetes diagnosis sometime after their baseline visits (incident cases).

**Table 8:** Clinical characteristics table stratified by type 2 diabetes at baseline visit

	Case inclusion	Case exclusion	Control inclusion	Control exclusion	*p*
n	13286	4029	478864	6279	
Age (mean (SD))	60.32 (6.75)	59.37 (7.33)	56.40 (8.10)	56.32 (8.20)	<0.001
BMI (mean (SD))	31.81 (5.90)	31.13 (6.06)	27.26 (4.67)	29.12 (5.51)	<0.001
Glucose (median [IQR])	6.42 [5.28, 8.50]	7.64 [5.46, 11.30]	4.91 [4.59, 5.27]	5.36 [4.79, 6.70]	<0.001
Glycated hemoglobin, HbA1c (median [IQR])	49.50 [43.10, 57.60]	58.20 [48.90, 69.10]	35.10 [32.70, 37.50]	40.70 [36.00, 51.00]	<0.001
Years since type 2 diabetes diagnosis (median [IQR])	4.05 [1.82, 7.16]	NA [NA, NA]	-6.27 [-9.09, -3.31] (Incident cases)	NA [NA, NA]	<0.001
Sex male (%)	8426 (63.4)	2370 (58.8)	215111 (44.9)	3198 (50.9)	<0.001
Family history of diabetes (%)	6219 (46.8)	1869 (46.4)	103336 (21.6)	2363 (37.6)	<0.001
Family history of heart disease (%)	6870 (51.7)	2005 (49.8)	217257 (45.4)	2900 (46.2)	<0.001
Family history of hypertension (%)	6565 (49.4)	1927 (47.8)	234153 (48.9)	3185 (50.7)	0.009
Hypertension (%)	10406 (78.5)	3120 (77.5)	140044 (29.3)	3275 (52.5)	<0.001
Hyperlipidemia (%)	11038 (83.4)	3232 (80.4)	82921 (17.4)	2964 (48.3)	<0.001
Atrial fibrillation (%)	345 (2.6)	84 (2.1)	4511 (0.9)	65 (1.0)	<0.001
Hypertrophic cardiomyopathy (%)	10 (0.1)	3 (0.1)	185 (0.0)	3 (0.0)	0.13
Heart failure (%)	380 (2.9)	201 (5.0)	1981 (0.4)	63 (1.0)	<0.001
On oral diabetes medication (%)	9057 (69.3)	1975 (49.4)	3910 (0.8)	1361 (22.3)	<0.001
On insulin (%)	1371 (10.4)	2672 (67.0)	481 (0.1)	1243 (19.9)	<0.001
Started insulin within 1 year of diagnosis (%)					<0.001
No	11642 (94.9)	2072 (59.3)	5988 (95.5)	2280 (69.7)	
Yes	508 (4.1)	1401 (40.1)	193 (3.1)	932 (28.5)	
Do not know	106 (0.9)	21 (0.6)	79 (1.3)	42 (1.3)	
Prefer not to answer	11 (0.1)	3 (0.1)	10 (0.2)	17 (0.5)	

The negative values of “Years since type 2 diabetes diagnosis” were attributed to future instances (incident cases).

**Figure 13:**
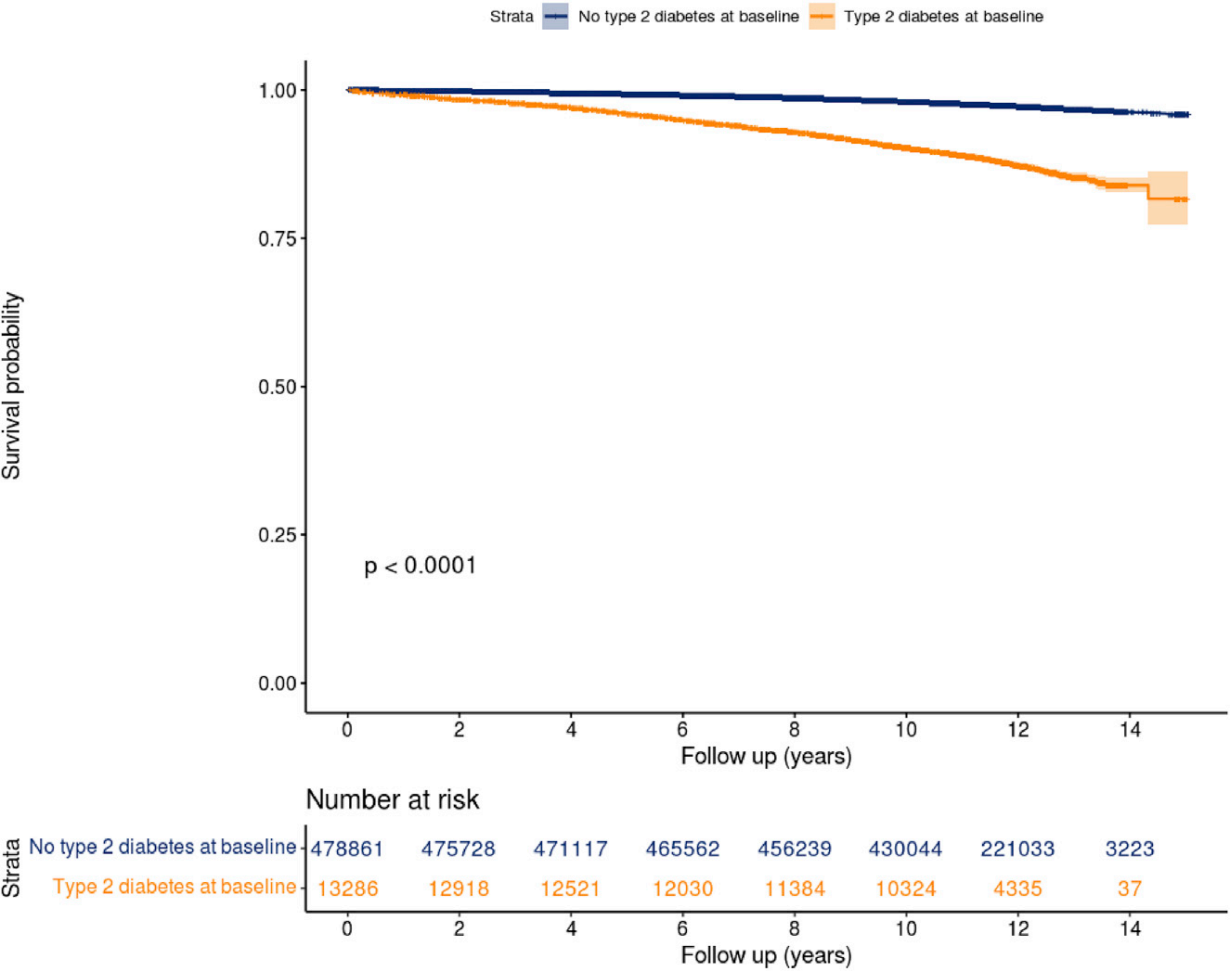
Kaplan-Meier plot for new-onset heart failure stratified by type 2 diabetes status at baseline The between-group difference in survival was assessed by log-rank test.

In the survival analysis on new-onset heart failure (outcome of interest) between participants with or without type 2 diabetes at baseline, user would obtain a Kaplan-Meier plot similar to Figure 13. Participants with type 2 diabetes at baseline were more likely to have heart failure. The between-group difference was assessed by log-rank test with *p*<0.0001 in the data analyzed.

### Case-control matching with MatchIt

**Figure 14:**
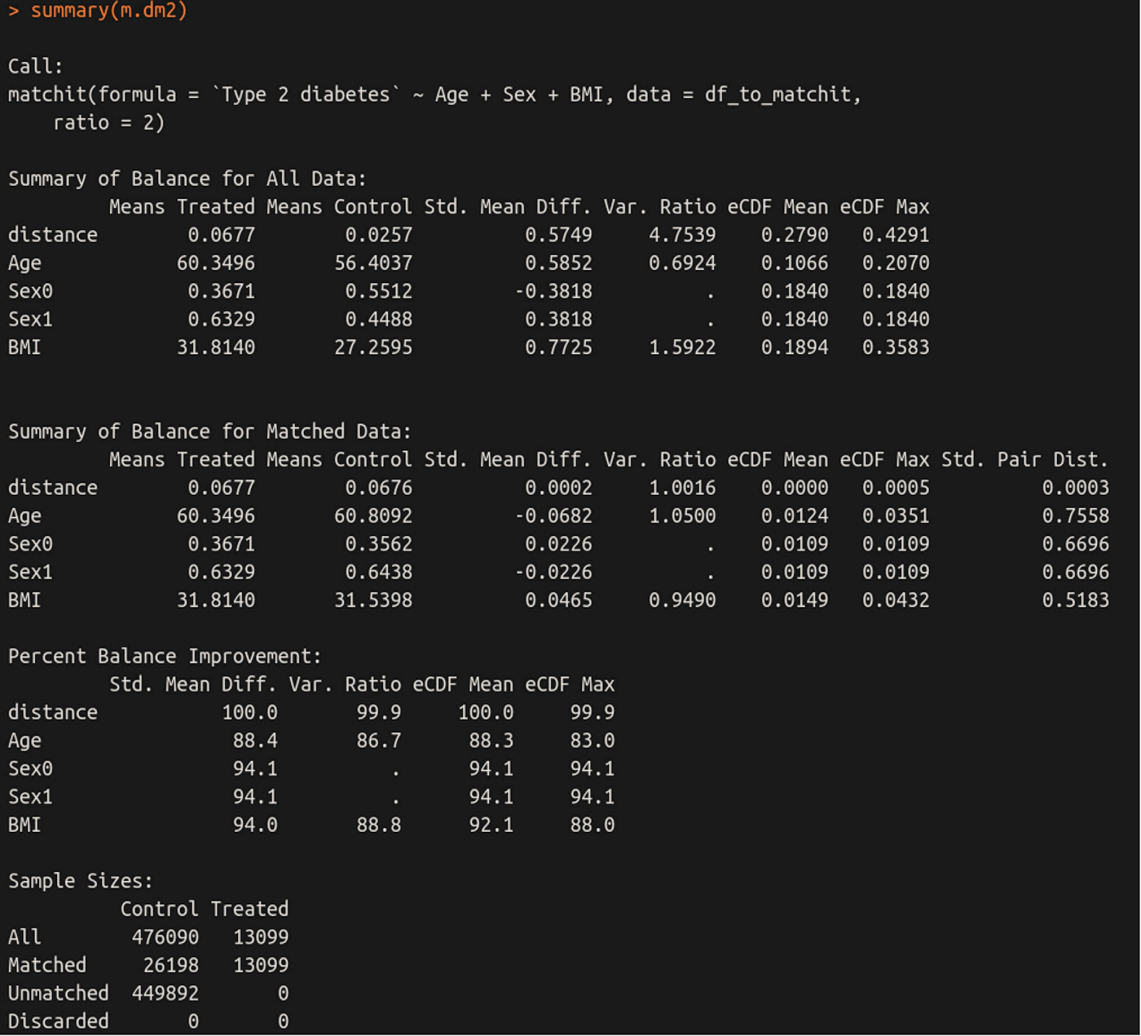
Result summary on case-control matching for type 2 diabetes status at baseline

Following up with the type 2 diabetes phenotype we created, it can be seen that case-control ratio is unbalanced. It can also be seen that the controls were younger, more likely to be female and had lower body mass indices compared to the cases (Table 8). We used the MatchIt package to match two controls to each case by these three covariates. After matching users should see these variables are balanced in cases and controls (Figure 14).

## Limitations

Phenotyping of a heterogenous resource such as UK Biobank can be challenging due to multiple data origins compounded with varying coverage both in terms of time and individuals. In the current protocol we demonstrate how to use the ukbpheno package to define health-related outcomes and we presented possible analyses with regard to the phenotyping process.

With the ukbpheno package, the case/control status is determined by presence of diagnosis records. In the case of contradictory records, an individual might be classified with theoretically mutually exclusive diagnoses. Ad-hoc analyses would be needed to refine the phenotypes of these individuals should such precision be required.

It is also of note that, while the users can assign different weights to different data sources by adjusting minimum instance filter in the setting, it is not possible to directly weight on individual codes which could be important for a certain phenotype. The limitation could be circumvented by an implementation on the definition level (separating the codes in various definitions). It is important to recognize that there is no gold-standard for many of the health-related outcomes. Users should decide on their definitions based on the study questions at hand.

## Troubleshooting

### Problem 1

Unable to download/decrypt the main dataset (step1).

### Potential solution

Ensure the most updated project key which is valid for one year after initiation.

### Problem 2

Unable to download data from Data Portal (step 2).

### Potential solution

Request the relevant fields listed in step 2 via the UK Biobank Access Management System (UK Biobank, 2022a).

### Problem 3

Error: Failed to install ‘ukbpheno’ from GitHub: installation of package “xx” had non-zero exit status (step 5).

### Potential solution

Installation of the dependent package “xx” was not successful. Find the error message on console, resolve the error and restart the installation. Possible reason of error includes “dependency “xx” is not available (for R version x.x.x) “, which may be solved by specifying the version of package compatible for the corresponding R version.

### Problem 4

Certain codes are missing after reading in the definition table (step 9).

### Potential solution

The package drops codes that are not available in data. If a certain code is dropped not due to this reason, check for special characters (accented characters are not recognized) and make sure codes are comma separated.

### Problem 5

Data processing step fails (step 10).

### Potential solution

Run “get_all_varnames()” with the processed definition table and meta-data file to check if but some required fields are missing in the main dataset. Either remove the missing fields from definition table or allow missing fields (set “allow_missing_fields” flag to TRUE) in the “harmonized_ukb_data()”.

## Resource availability

### Lead contact

Further information and requests for resources should be directed to the lead contact, Ming Wai Yeung (m.w.yeung@umcg.nl).

### Materials availability

This study did not generate new unique reagents.

## Data Availability

Data used and generated are listed in the key resources table. The UK Biobank data are open to bona-fide researchers and accessible with an approved research project. Application guideline for the UK Biobank data can be found at https://www.ukbiobank.ac.uk/enable-your-research/apply-for-access. The code supporting the current study is available at the ukbpheno repository https://github.com/niekverw/ukbpheno/blob/master/inst/workflow_example/run_example.R.
